# Ketones improves Apolipoprotein E4-related memory deficiency via sirtuin 3

**DOI:** 10.18632/aging.102070

**Published:** 2019-07-07

**Authors:** Junxiang Yin, Megan Nielsen, Shiping Li, Jiong Shi

**Affiliations:** 1 Barrow Neurological Institute, St. Joseph Hospital and Medical Center, Dignity Health Organization, Phoenix, AZ 85013, USA; 2 School of Life Sciences, Arizona State University, Tempe, AZ 85257, USA; 3 Department of Neurology, The Second Hospital of Hebei Medical University, Shijiazhuang, Hebei 050000, China; 4 Advanced Innovation Center for Human Brain Protection, Capital Medical University, Beijing 100160, China; 5 China National Clinical Research Center for Neurological Diseases, Beijing Tiantan Hospital, Capital Medical University, Beijing 100160, China

**Keywords:** apolipoprotein E4, Alzheimer’s disease, sirtuin

## Abstract

Background: Apolipoprotein E4 (ApoE4) is the major genetic risk factor of Alzheimer’s disease (AD). ApoE4 carriers have cerebral hypometabolism which is thought as a harbinger of AD. Our previous studies indicated ketones improved mitochondria energy metabolism via sirtuin 3 (Sirt3). However, it is unclear whether ketones upregulate Sirt3 and improve ApoE4-related learning and memory deficits.

Results: Ketones improved learning and memory abilities of ApoE4 mice but not ApoE3 mice. Sirt3, synaptic proteins, the NAD^+^/ NADH ratio, and ATP production were significantly increased in the hippocampus and the cortex from ketone treatment.

Methods: Human ApoE3 and ApoE4 transgenic mice (9-month-old) were treated with either ketones or normal saline by daily subcutaneous injections for 3 months (ketones, beta-hydroxybutyrate (BHB): 600 mg/kg/day; acetoacetate (ACA): 150 mg/kg/day). Learning and memory ability of these mice were assessed. Sirt3 protein, synaptic proteins (PSD95, Synaptophysin), the NAD^+^/ NADH ratio, and ATP levels were measured in the hippocampus and the cortex.

Conclusion: Our current studies suggest that ketones improve learning and memory abilities of ApoE4 transgenic mice. Sirt3 may mediate the neuroprotection of ketones by increasing neuronal energy metabolism in ApoE4 transgenic mice. This provides the foundation for Sirt3’s potential role in the prevention and treatment of AD.

## INTRODUCTION

Apolipoprotein E4 (ApoE4) is found in 25% of the population, and in 60% of Alzheimer’s disease (AD) patients. Cerebral hypometabolism is associated with decreased memory ability and is believed to be a diagnostic biomarker for cognitive impairment at preclinical stages of AD [1–5]. Individuals with ApoE4 have reduced glucose metabolism even in young adulthood in specific brain regions [6–11]. Studies also found that ApoE4 was also associated with reduced ATP levels in the cerebral cortex in mouse models [12–15].

Our previous studies found that peripheral administration of ketones significantly reduced amyloid burden and greatly improved learning and memory ability in a symptomatic model of APP transgenic mice [16]. Sirt3 was involved in the pathogenesis of AD and was downregulated by Aβ [17, 18]. Further studies discovered that the Sirt3-related energy pathway played a critical role in neuronal hypometabolism [18]. For example, Sirt3 mediates the neuroprotective effect of ketones in an ischemia mouse model of middle cerebral artery occlusion [19]. These findings support the concept that ketones are not only metabolites [20, 21], but they are also endogenous neuroprotective agents [16, 22]. However, it is not fully understood whether ketones upregulate Sirt3 and improve learning and memory abilities of ApoE4 transgenic mice. We hypothesize that Sirt3 mediates the neuroprotection of ketones in ApoE4 transgenic mice. In this study, 9-month-old human ApoE3 and ApoE4 transgenic mice were treated with either ketones or normal saline by daily subcutaneous injections for 3 months. We assessed learning and memory abilities, followed by measuring levels of Sirt3, synaptic proteins, the NAD^+^/ NADH ratio, and ATP production in the hippocampus and the cortex.

## RESULTS

### Ketones improved learning and memory abilities of ApoE 4 mice

**Figure 1:**
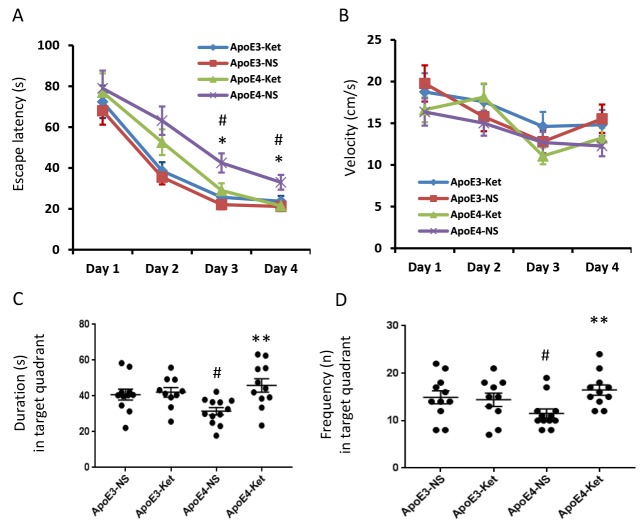
**Ketones improve learning and memory abilities of ApoE 4 mice.** ApoE4 and ApoE3 mice were randomly divided into ketone treatment (Ket) or normal saline (NS) groups. After three months of treatment, all mice were tested in the Morris water maze. (**A**) Escape latency (s); (**B**) Velocity (cm/s); (**C**) Duration in the target quadrant (s); (**D**) Frequency in the target quadrant. N= 10–11 per group, #p<0.05, ApoE3-NS vs. ApoE4-NS. *p<0.05, **p<0.01, ApoE4-NS vs. ApoE4-Ket.

After 3-month treatment of ketones or normal saline, all mice were tested for their learning and memory abilities in the Morris water maze. The escape latency measures how fast the mouse can find the platform to escape out of the water maze. In saline treatment groups, the escape latency of ApoE 4 mice was longer than that of ApoE 3 after two days of training (p< 0.05, Figure 1A). There was no difference in swimming speed (Figure 1B). Ketone treatment improved the escaped latency in ApoE4 mice compared to ApoE4 mice receiving normal saline treatment (p< 0.05, Figure 1A). Ketones however didn’t improve the swimming speed (Figure 1B). Similar results were found in the probe test after four days of training. ApoE4 mice who received normal saline had the shortest duration and the least frequency in the target quadrant; while ApoE4 mice who received ketone treatment had the longest duration and the most frequency in the target quadrant (Figure 1D, 1E). This data indicated that ApoE4 mice had learning and memory impairment compared to ApoE3 mice. Ketone treatment improved learning and memory abilities of ApoE4 mice.

### Ketones increased the NAD^+^/ NADH ratio and ATP levels in ApoE 4 mice

**Figure 2:**
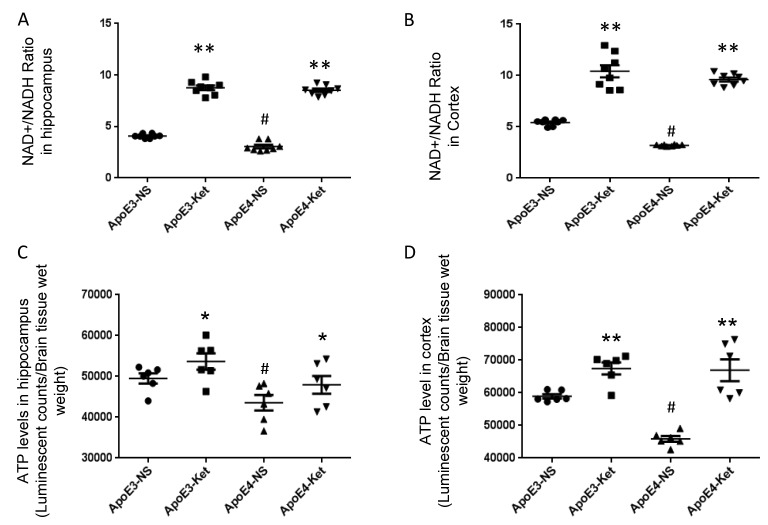
**Ketones increase the NAD^+^/ NADH ratio and ATP levels in ApoE 4 mice.** ApoE4 and ApoE3 mice were treated with either ketones or normal saline for three months. Fresh hippocampal and cortical tissues were collected to measure NAD, NADH and ATP levels. (**A**) the NAD^+^/ NADH ratio in the hippocampus; (**B**) the NAD^+^/ NADH ratio in the cortex; (**C**) ATP levels in the hippocampus; (**D**) ATP levels in the cortex. N= 6–8 per group, # p<0.05, ApoE3-NS vs. ApoE4-NS. * p<0.05, ** p<0.01, ApoE3-NS vs. ApoE3-Ket, ApoE4-NS vs. ApoE4-Ket.

Since Sirt3 is NAD^+^-dependent, we tested the effect of ketones on NAD^+^/ NADH ratio in mouse fresh hippocampal and cortical tissues. After ketone treatment, the NAD^+^/NADH ratio was significantly increased in the hippocampus (ApoE3-NS: 4.1±0.1 vs. ApoE3-Ket: 8.8±0.2; ApoE4-NS: 3.1±0.2 vs. ApoE4-Ket: 8.5±0.2; NS vs. Ket, *p*<0.01, Figure 2A) and the cortex (ApoE3-NS: 5.4±0.1 vs. ApoE3-Ket: 10.4±0.6; ApoE4-NS: 3.2±0.1 vs. ApoE4-Ket: 9.6±0.2; NS vs. Ket, p<0.01, Figure 2B) in both ApoE3 and ApoE4 mice. We further measured ATP levels to evaluate the energy metabolism. Similar results were found on the change in ATP levels after ketone treatment in the hippocampus (ApoE3-NS: 49533±1239 vs. ApoE3-Ket: 53688±1990; ApoE4-NS: 43541±1902 vs. ApoE4-Ket: 47946±2177; NS vs. Ket, p<0.05, Figure 2C) and the cortex (ApoE3-NS: 58923±1663 vs. ApoE3-Ket: 67486±1846; ApoE4-NS: 45902±880.4 vs. ApoE4-Ket: 66935±3322; NS vs. Ket, p<0.01, Figure 2D) in both ApoE3 and ApoE4 mice.

**Figure 3:**
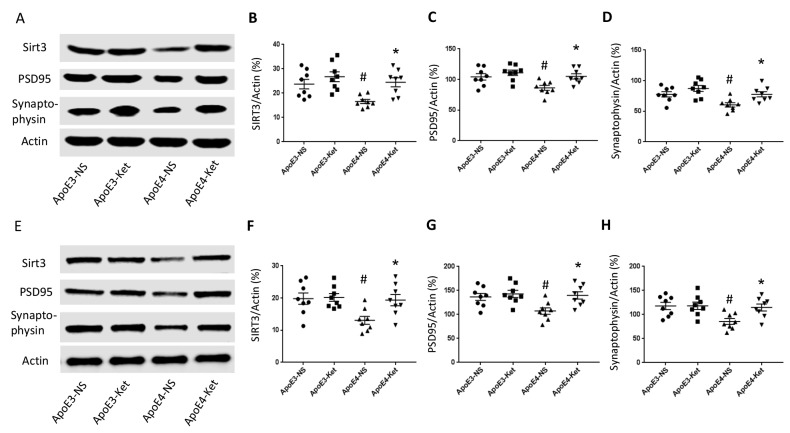
**Ketones upregulate Sirt3, PSD95 and Synaptophysin in ApoE 4 mice.** ApoE4 and ApoE3 mice were treated with either ketones or normal saline for three months. Fresh hippocampal and cortical tissues were collected and homogenized for western blot. (**A**–**D**) Proteins levels were measured in the hippocampus: (**A**) Representative western blot bands for Sirt3, PSD95, Synaptophysin, and Actin were shown. (**B**) Sirt3, (**C**) PSD95 and (**D**) Synaptophysin levels were normalized with Actin. (**E**–**H**) Proteins levels were measured in the cortex: (**E**) Representative western blot bands for Sirt3, PSD95, Synaptophysin and Actin were shown. (**F**) Sirt3, (**G**) PSD95 and (**H**) Synaptophysin levels were normalized with Actin. N=8 per group, # p<0.05, ApoE3-NS vs. ApoE4-NS; * p<0.05, ApoE4-NS vs. ApoE4-Ket.

### Ketones upregulated Sirt3, PSD95 and Synaptophysin in ApoE 4 mice

Since synaptic integrity is critical for normal learning and memory ability, we measured the levels of post-synaptic density protein 95 (PSD95), major synaptic vesicle protein (Synaptophysin) and Sirt3 in the hippocampus and the cortex after 3 months of ketone treatment. The treatment increased Sirt3, PSD95 and Synaptophysin levels in ApoE4 mice but not in ApoE3 mice. In the hippocampus (Figure 3A-D), Sirt3 levels (vs. Actin) were 23.7±2.0 (ApoE3-NS), 26.8±2.0 (ApoE3-Ket), 16.6±0.9 (ApoE4-NS), and 24.5±1.8 (ApoE4-Ket); PSD95 levels (vs. Actin) were 104.3±5.2 (ApoE3-NS), 111.0±4.4 (ApoE3-Ket), 86.3±4.0 (ApoE4-NS), and 104.1±4.3 (ApoE4-Ket) and Synaptophysin levels (vs. Actin) were 77.7±4.3 (ApoE3-NS), 87.3±4.9 (ApoE3-Ket), 60.5±3.6 (ApoE4-NS), and 76.7±4.2 (ApoE4-Ket). Similarly in the cortex (Figure 3E-H), Sirt3 levels (vs. Actin) were 19.8±1.8 (ApoE3-NS), 20.2±1.2 (ApoE3-Ket), 13.1±1.3 (ApoE4-NS), and 19.4±1.7 (ApoE4-Ket); PSD95 levels (vs. Actin) were 136.5±7.3 (ApoE3-NS), 142.8±7.4 (ApoE3-Ket), 107.2±6.8 (ApoE4-NS), and 139.8±7.7 (ApoE4-Ket) and Synaptophysin levels (vs. Actin) were 117.8±7.4 (ApoE3-NS), 118.2±7.6 (ApoE3-Ket), 85.6±6.0 (ApoE4-NS), and 114.7±7.3 (ApoE4-Ket).

## DISCUSSION

In this study, our data showed that ketones enhanced the NAD^+^/ NADH ratio to increase Sirt3 expression, this led to improved ATP production, better synaptic integrity and finally improved the learning and memory ability of ApoE4 mice. Consistent with previous studies, our data suggest that Sirt3 mediates the neuroprotective effects of ketones by increasing neuronal energy metabolism in ApoE4 transgenic mice.

Literature has shown that regional cerebral glucose hypometabolism plays a key role in AD pathogenesis and is correlated with the disease severity [23, 24]. Cerebral hypometabolism is one of the early signs of AD and is thought to be a predictor of disease progression [1–3]. ApoE4 is the major genetic risk factor of AD. Studies also find that ApoE4 is associated with reduced ATP levels in the cerebral cortex in mouse models [12–15]. Our previous studies showed that Sirt3 played a critical role in the pathological change of AD and was significantly reduced by Aβ in our in vitro and in vivo experiments [17, 18]. The Sirt3-related energy pathway is involved in neuronal hypometabolism [18]. Sirt3 is NAD^+^-dependent. It is critical for ATP production in mitochondria. Knocking down Sirt3 reduces ATP production while overexpressing Sirt3 increases it [18]. Ketones, by enhancing the NAD^+^/ NADH ratio, regulate Sirt3 which in turn improves ATP production in mitochondria. Particularly in ApoE4 mice, ketone treatment enhances the levels of NAD^+^, ATP, Sirt3 and synaptic proteins. This preservation in synaptic integrity translates into the improvement in learning and memory abilities. This suggests Sirt3 in addition to improvement in mitochondrial energy may also serve to protect synapses.

Ketones can serve as an important energy substrate under starvation. Its routine use has been suggested to help professional athletes to improve their performance, but how ketones regulate the metabolism of skeletal and cardiac muscles during exercise of varying intensity and duration remains unknown [25, 26]. Our data didn’t show a change in swimming speed in any of the groups, with or without treatment. But we only tested them for a short period of time. A more detailed study with various time and exercise intensity will be warranted.

In summary, we have shown that ketones protect ApoE4 related learning and memory impairment via Sirt3 regulation. This will shed lights on the development of new therapeutic agents.

## METHODS

### ApoE 4 and ApoE 3 transgenic mice

Transgenic mice carrying either human ApoE4 or ApoE3 allele were purchased from Taconic Biosciences (Rensselaer, NY). These transgenic lines have been shown to transcribe and express appropriate human ApoE3 and ApoE4 protein in the brain, the liver, and other tissues without contamination of the endogenous mouse ApoE gene. We used 9-month-old homozygous mice (littermates) for our experiments. ApoE3 mice and ApoE4 (10-11 animals/ group) were treated with ketones or control saline by daily subcutaneous injections from 9 months of age (ketones, beta-hydroxybutyrate (BHB): 600 mg/kg/day; acetoacetate (ACA): 600 mg/kg/day; ACA: 150 mg/kg/day). All mice were labeled with serial numbers and were blinded to the person who tested their behavior or Western blot etc. After behavioral tests, animals were euthanized. The brain was processed for further analysis.

All mice were housed in a temperature and humidity-controlled vivarium, kept on a 12-hour dark/light cycle, and had free access to food and water. All experimental procedures were approved by the Institutional Animal Care and Use Committee of the Barrow Neurological Institute and performed according to the Revised Guide for the Care and Use of Laboratory Animals.

### Morris water maze

Spatial learning was assessed by the Morris Water Maze (MWM) task adapted for mice [16]. Briefly, each mouse was introduced into the circular pool and allowed to swim. The time (escape latency) required to reach the platform located in northeast quadrant, as well as the swimming speed was recorded in each trial. Once the mouse located the platform, it was permitted to remain on it for 10 seconds. If the mouse did not locate the platform within 120 seconds, it was placed on the platform for 10 seconds. The mouse was given four trials per day for 4 days with an inter-trial interval of 20 minutes. Each trial was initiated by randomly placing an animal in one of the four starting locations. Escape latency and swimming speed were collected and analyzed using EthoVision® 3.1 tracking software (Noldus Information Technology Inc., Leesburg, VA). On the 5^th^ day, a single probe trial was carried out. In this trial, the platform was removed and each mouse was placed from southwest quadrant of the pool and allowed to swim for 120 seconds. The time spent in the target quadrant (northeast) was collected and calculated using EthoVision® 3.1 tracking software.

### Brain tissue collection

After 3-month treatment, mice were placed in a chamber with 5% isoflurane for 5 min, and decapitated when fully sedated. Fresh brain tissues (hippocampus and temporal cortex) were isolated and collected immediately on ice. For western blot, brain tissues (hippocampus and cortex) were homogenized and sonicated with ice-old RIPA buffer (#9806, Cell Signaling, Danvers, MA) with protease inhibitor cocktail (#p8340, Sigma-Aldrich, St.Louis, MO) on ice. Total proteins were collected after centrifugation at 4°C (19000g, 20mins) and saved in −80°C for western blot. For ATP and NAD+/NADH measurements, fresh temporal brain tissues were collected. ATP levels, total NAD+ and NADH were tested according to the assay protocols.

### Western blot

Fifty μg total proteins were used for western blotting. Primary antibodies were the following: anti-Sirt3 (#5490S, Cell Signaling Technology Inc., Danvers, MA), anti-postsynaptic density protein 95 (PSD-95, #3450, Cell Signaling Technology Inc.), anti-synaptophysin (#12270, Cell Signaling Technology Inc.), anti-β-actin (Santa Cruz, Dallas, TX), IRDye 800CW and IRDye 680CW antibodies (LI-COR Biosciences, Lincoln, NE). Immunoreactivity signals were quantified using Odyssey CLx. Protein levels were presented by a percentage relative to β-actin, an internal control.

### ATP measurements

Fresh mouse temporal brain tissue (20 mg) was collected and homogenized in 200 ul 2M perchloric acid on ice. Samples were kept on ice for 30 minutes. After the samples were centrifuged, the supernatant was collected and diluted the volume to 1000ul with ATP assay buffer. Then, the supernatant was transferred into two new 500ul tubes. Supernatant were neutralized to a pH between 7.0 and 7.6, and excess perchloric acid was precipitated with ice-cold potassium hydroxide (2M). After repeating the neutralization and centrifugation process, new supernatant was collected for ATP assay. ATP levels were tested using a Luminescent ATP detection assay kit (#ab83355, Abcam, Cambridge, MA) according to the manufacturer’s protocol.

### NAD^+^ and NADH measurements

Fresh mouse temporal brain tissue (20 mg) was collected and homogenized in 400ul NAD^+^/ NADH extract buffer on ice. After the samples were centrifuged, the supernatant was collected into a new tube and deproteinized with perchloric acid (4M). After a second centrifuge and second supernatant was transferred into a new tube, the supernatant was neutralized to a pH between 7.0 and 7.6 with ice-cold potassium hydroxide (2M). Then, the samples were centrifuged and the supernatant was collected for total NAD^+^ and NADH assay. Total NAD^+^ and NADH were tested using an NAD^+^/NADH assay kit (#ab65348, Abcam) according to the manufacturer’s protocol. NAD^+^/NADH ratio was calculated based on the value of total NAD^+^ and NADH (NAD^+^ = total NAD^+^ – NADH).

### Statistics

We applied ANOVA with post-hoc Tukey’s test to compare values across multiple groups by using GraphPad Prism version 7.03 and Pearson’s correlation analysis for correlation analyses by using IBM SPSS statistics version 23. Statistical significance was set at p< 0.05.
